# Dynamics of Peripheral Blood Immune Cells during the Perioperative Period after Digestive System Resections: A Systematic Analysis of the Literature

**DOI:** 10.3390/jcm12020718

**Published:** 2023-01-16

**Authors:** Markus Bo Schoenberg, Yongsheng Han, Xiaokang Li, Xinyu Li, Julian Nikolaus Bucher, Nikolaus Börner, Dominik Koch, Markus Otto Guba, Jens Werner, Alexandr V. Bazhin

**Affiliations:** 1 Department of General, Visceral, and Transplant Surgery, Ludwig-Maximilians-University Munich, 81377 Munich, Germany; 2 German Cancer Consortium (DKTK), Partner Site Munich, 81377 Munich, Germany; 3 Medical Center Gollierplatz, 80339 Munich, Germany; 4 Transplantation Center Munich, Hospital of the LMU, Campus Grosshadern, 81377 Munich, Germany

**Keywords:** digestive system tumors, perioperative period, peripheral blood immune cells

## Abstract

An operation in itself is a kind of trauma and may lead to immunosuppression followed by a bounce back. Not many studies exist that describe dynamics of the distribution of peripheral blood (PB) immune cells during the perioperative period. Considering this scarcity, we aggregated the data on the dynamics of immune cells in patients with digestive system resections during the perioperative period and the relationship with short- and long-term prognoses. By the systematic retrieval of documents, we collected perioperative period data on white blood cells (WBC), lymphocytes, neutrophil–lymphocyte ratio (NLR), CD4^+^ T cells, CD8^+^ T cells, helper T cells (Th), B cells, natural killer cells (NK), dendritic cells (DCs), regulatory T cells (Tregs), regulatory B cells (Bregs), and Myeloid derived suppressor cells (MDSC). The frequency and distribution of these immune cells and the relationship with the patient’s prognosis were summarized. A total of 1916 patients’ data were included. Compared with before surgery, WBC, lymphocytes, CD4^+^ cells, CD8^+^ T cells, MDSC, and NK cells decreased after surgery, and then returned to preoperative levels. After operation DCs increased, then gradually recovered to the preoperative level. No significant changes were found in B cell levels during the perioperative period. Compared with the preoperative time-point, Tregs and Bregs both increased postoperatively. Only high levels of the preoperative and/or postoperative NLR were found to be related to the patient’s prognosis. In summary, the surgery itself can cause changes in peripheral blood immune cells, which might change the immunogenicity. Therefore, the immunosuppression caused by the surgical trauma should be minimized. In oncological patients this might even influence long-term results.

## 1. Introduction

Previous studies have shown that immunosuppression caused by surgery may lead to tumor recurrence, deterioration, and metastasis. The triggers involve inflammation, ischemia-reperfusion injury, activation of the sympathetic nervous system, and increased cytokine release. Furthermore, immunosuppression and upregulation of adhesion molecules may also create favorable conditions for tumor metastasis [1]. This has been reported on tumors of the digestive system such as stomach cancer, hepatocellular carcinoma, and colorectal cancer [2,3,4]. Additionally, in some related work, it has been suggested that this immunological change is related to the patient’s prognosis, and can even be considered a biological marker to predict the survival of patients [5,6,7,8]. With the dawn of novel immunotherapeutic agents for neoadjuvant and adjuvant treatment, detailed experimental analyses of perioperative immune cell distribution are an emerging field.

T-cells in general can kill tumor cells directly (cytotoxic T-cells) or assist (helper T-cells) other lymphocytes to exert immunological activity. This does not only lead to immune activating but in some cases conversely to immunosuppressive effects (T regulatory cells) [9]. B cells’ primary function is to secrete antibodies that mediate humoral immune responses. A newly discovered subpopulation of B-cells are regulatory B cells (Breg) [10]. The main mechanism of Bregs is to promote the development of Tregs and inhibit the effector Th cells and cytotoxic T lymphocytes by secreting IL-10 [11]. Another important group are NK-cells which can directly kill virus infected cells or tumor cells [12]. DCs are the most potent antigen-presenting cells derived from bone marrow precursors which can express co-stimulatory molecules and higher major histocompatibility complexes (MHC). They play an important role in the initiation and regulation of immune system responses [13]. Takahashi et al. divided DCs into two functionally heterogeneous subgroups: DC1 (CD11c^+^ DCs, known as myeloid DCs, which stimulate naïve T cells to protect against cancer) and DC2 (CD11c^−^ DCs, known as lymphoid DCs, which activate Th2 cells which generate IL-4) [14].

Up to now, no systematic analysis exists that describes the dynamics of immune cells perioperatively. Therefore, the aim of this work was to provide a comprehensive systematic overview over the perioperative major immune reaction. We aimed to put the results into an oncological context.

## 2. Materials and Methods

The search terms (“Immune System”) AND “Digestive System Neoplasms” AND “Perioperative Period” were used to search PubMed. The last time point for the search was November 2022. The retrieval strategy was first to browse the titles and abstracts of the publications, then we selected the relevant ones, and read the full text. References of retrieved full-texts were additionally scanned for relevant publications to reduce omissions. We used prospectively created inclusion and exclusion criteria to focus the literature for this analysis:

**Literature inclusion criteria:** (1) Research type: clinical research; (2) Research object: human; (3) Research content: circulating immune cells; (4) Literature languages: English. (5) Perioperatively follow-up time-frame: from before the operation to 1 year after surgery.

**Literature exclusion criteria:** (1) Published before 2000 or not in English; (2) Clinical trials, studies of therapy, etc.; (3) Case reports, meta-analyses or reviews; (4) Animal research; (5) non-digestive system tumor; (6) Only preoperative data; (7) Research on genes, non-immunological proteins, etc.; (8) No surgery performed.

To unify the results, we divided postoperative time-points into the following three timeframes: Within 7 days after surgery, between 7 days after surgery up until 3 months after surgery and from 3 months after surgery to 1 year after the operation.

## 3. Results

### 3.1. Study Characteristics

**Figure 1:**
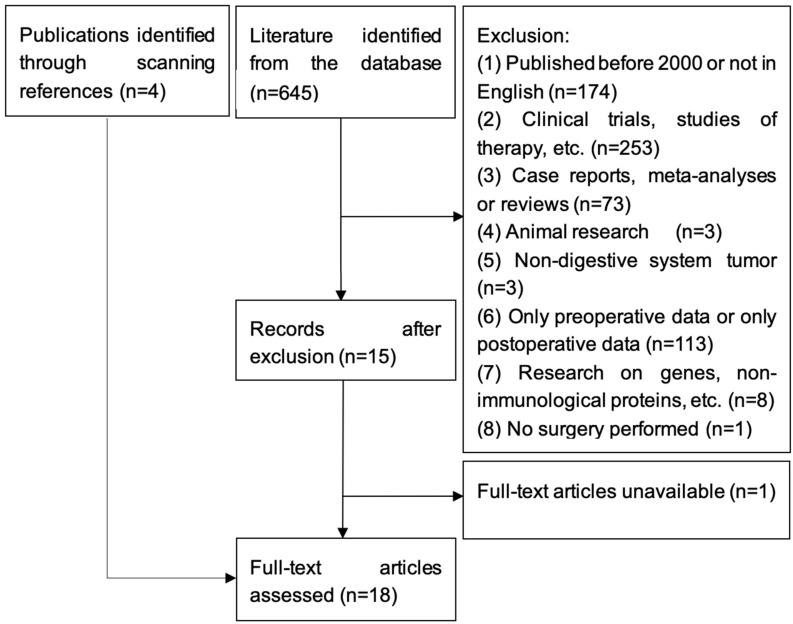
Flow-chart of study selection.

Following the above-described search strategy, 645 related publications were identified (Figure 1). According to the exclusion criteria, 630 articles were excluded. Three additional publications could be identified through scanning the references of included publications. One paper had to be excluded because no full-text was available [15]. Finally, 18 studies met the predefined inclusion criteria and were included in this analysis. The study selection flow-chart is depicted in Figure 1.

**Figure 2:**
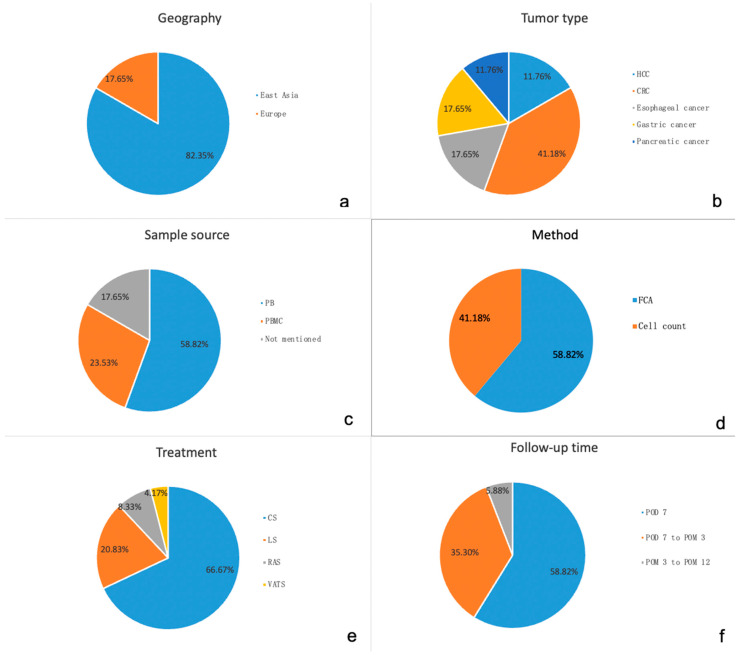
Characteristics of studies included in the analysis. (**a**): geographic distribution of publications; (**b**): classification of digestive system tumors; (**c**): source of the specimen; (**d**): detection methods; (**e**): surgical methods; (**f**): postoperative follow-up time. (Abbreviations: CRC: Colorectal cancer; HCC: Hepatocellular carcinoma; PB: Peripheral blood; PBMC: Peripheral blood mononuclear cells; FCA: Flow cytometry analysis; CS: Conventional surgery; LS: Laparoscopic; RAS: Robot-assisted surgery; VATS: Video-assisted thoracoscopic surgery; POD: Postoperation day; POM: Postoperation month).

Eighteen studies (N = 1916 patients) examined changes in immune cells in digestive system tumors perioperatively and were included in this analysis. As can be seen in Figure 2a, most of the studies (n = 15; 83.33%) were performed in East Asia, especially in China and Japan [14,16,17,18,19,20,21,22,23,24,25,26,27,28,29]. Three studies were conducted in Europe (n = 3; 16.67%) [30,31,32]. The most common digestive system tumor type of included studies was colorectal cancer (CRC) (n = 7; 41.18%) [17,19,20,23,26,30,31]. Three studies offered information on gastric cancer or esophageal cancer (16.67%) [16,18,22,25,27,32]. Samples from pancreatic tumor patients were measured in two studies (11.11%) [14,24]. Hepatocellular carcinoma (HCC) patients were investigated in three studies (16.67%) (Figure 2b) [14,21,24,28,29] As shown in Figure 2c, 10 (55.56%) studies measured immune cells in fresh peripheral blood (PB) samples [14,16,18,19,20,23,26,27,31,32]. Five (27.78%) studies measured peripheral blood monocyte cells isolated from the blood for measurements [17,22,25,28,29]. Three studies did not describe the source of samples in detail (n = 3; 16.67%) [21,24,30]. Flow cytometry analysis was used in more than half of the studies (n = 11; 61.11%) [14,17,18,19,20,22,25,27,28,29,31] PB cell count analyses (as performed in the routine laboratory) were used in the remaining studies (n = 7; 38.89%) (Figure 2d) [16,21,23,24,26,30,32]. As can be seen in Figure 2e, the most common treatment of patients in the included studies was conventional surgery (CS) (n = 17; 68.00%) [14,16,17,18,19,20,21,22,23,24,25,26,27,28,29,31,32]. Others are minimally invasive surgery, including laparoscopic surgery (n = 5; 20.00%) [25,26,30,31,32], robot-assisted surgery (n = 2; 8.00%) [19,30], and video-assisted thoracoscopic surgery (n = 1; 4.00%) [18] Postoperative follow-up times varied across publications. Data within 7 days after surgery were reported in n = 11 (57.83%) studies [17,18,19,22,23,25,28,29,30,31,32]. Between 7 days after surgery up until 3 months after surgery n = 7 (36.84%) publications reported results [16,20,21,24,26,27,29]. Lastly, data from 3 months after surgery to 1 year after the operation were found in 1 (5.30%) study [14]. These data are depicted in Figure 2f. In all the included publications, the farthest time-point after surgery was one year after surgery. Six (30%) studies were conducted retrospectively, five (27.78%) studies were prospective case control studies, three (16.67%) were prospective uncontrolled studies, and finally, four (22.22%) were randomized controlled trials.

### 3.2. Gastric Cancer

Perioperative immune cell changes were investigated in n = 333 gastric cancer patients (Table 1) [16,25,27]. These studies reported on the NLR, WBC, and subgoups of lymphocytes. Independently from each other, Fuji et al. and Takaya et al. could show that WBC counts increased in the immediate postoperative period but returned to preoperative levels at POD 7 and POM 1, respectively [25,27]. Furthermore, Fuji et al. described a decrease of Lymphocytes in the first 7 days after resection. This decrease was maintained at POD 7.

CD3^+^, CD4^+^, CD8^+^, CD57^+^, and HLA-DR^+^ cells decreased on POD 1, then returned to preoperative level on POD 7 [25]. Miyatani et al. described in their retrospective observational study different groups with pre- and postoperative NLR. According to them, a low NLR both before and after the operation was associated with the best 5-year survival of 92.8% [16]. They reported on patients who underwent CS, and calculated NLR preoperatively and within 3 months after the operation. The results showed that neither preoperative nor postoperative NLR alone was able to predict survival after gastrectomy. In combination, however, the 5-year survival rate of patients with a low preoperative and postoperative NLR was significantly better than with a high preoperative and/or postoperative NLR. After an analysis of the area under the curve, poor yet significant predictability was shown [16]. Generally, a low NLR is a direct result of low neutrophil or comparably high lymphocyte levels.

LS was only reported in 10 patients. Fuji et al. could show that although distributionally similar, Th1 function was better preserved after LS [25].

Confounding factors such as adjuvant chemotherapy was investigated by Myatani et al., where 62 of a total of 280 patients received adjuvant chemotherapy. No differences in NLR were found between the chemo and non-chemo group. The authors argue that adjuvant chemotherapy is generally induced 4–6 weeks after surgery. The measurement of the NLR was performed at Week 4 after the operation [16].

### 3.3. Hepatocellular Carcinoma

A total of 244 HCC patients were investigated according to their perioperative alteration of immune cells (Table 1). As shown in Table 1, Chen et al. and Lee at al. both investigated Tregs. The research by Chen et al. showed that a group of Tregs (defined as CD4^+^CD25^+^CD127^−^ in this publication) dramatically increased after resection in 36 HCC patients. This effect was highest on POD 7, but they did not report when it recovered to the preoperative level [28]. In contrast to that, Lee et al. reported that CD4^+^Foxp3^+^ Tregs remained unchanged by the operation [29].

**Figure 3:**
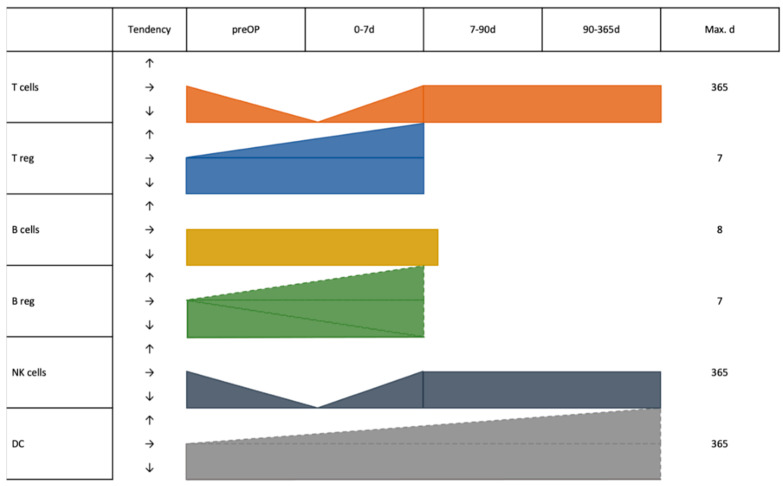
Graphical depiction of the dynamics of major immune cells after resection of gastrointestinal tumors. Dashed lines indicate contradictory results from different studies.

Chen et al. also examined Bregs (defined as CD19^+^IL-10^+^ cells) pre- and postoperatively in 36 HCC patients. Similar to Tregs, the frequency of peripheral Bregs significantly increased after tumor resection in 36 HCC patients, especially at POD 7 [28]. Chen et al. described in the discussion that the increase in postoperative Bregs can promote tumor metastasis and recurrence in HCC patients due to inhibition of immunity after resection [28] (Figure 3).

Only one publication reported on results concerning MDSC after liver resection because of HCC. MDSC frequency was significantly decreased at one month after resection. These results have not been confirmed by other publications yet [29].

At POM 1 Peng et al. calculated the ΔNLR (postoperative NLR–preoperative NLR = ΔNLR). The groups were divided into decreasing and increasing NLR. Patients with a decreasing NLR after surgery showed significantly better overall survival (OS) and recurrence-free survival (RFS, date of resection to recurrence or death from any cause) [21].

All operations were performed with a conventional open technique. Due to its nature, there is no neoadjuvant or adjuvant treatment against HCC. Of course, Hepatitis is able influence circulating immune cells. Chen et al. was able to show a weak correlation of Tregs and Bregs with the presence of hepatitis B. This means that the levels of Tregs were generally lower; however, the longitudinal changes remained [28].

### 3.4. Colorectal Cancer

A total of 953 CRC patients were investigated according to their perioperative immune cell distribution (Table 1). Kubo et al. measured the NLR at 3 time-points within 7 days perioperatively (preoperatively, POD 1, and POD 3) in 524 CRC patients. Subsequently, the NLR was divided into a high NLR group (high NLR at >1 time-point) and a low NLR group (high NLR at 0–1 time-points). The results revealed that a persistently increased NLR during the perioperative period served as an independent risk factor for both the cancer-specific survival (CSS, date of resection to death due to recurrence) and disease-free survival (DFS, date of resection to recurrence) after curative resection [23].

Ordemann et al. could show that WBC increased on POD 1, then returned to preoperative level on POD 7. This was confirmed by Leung et al. [26,31].

In the same study on lymphocytes, T cells, B cells, non-MHC restricted NK cells, NK cells, natural Killer-like T cells, cytotoxic T cell, and helper T cells, T cell activation decreased on POD 1, then returned to preoperative level on POD 8. Shibata et al. described NK cells, CTL, and Th cells, which decreased on POD 1 and increased again on POD 3 and POD 6 [19].

Shibata et al. also measured the B cells (defined as CD3^−^/CD19^+^) at four time-points within 7 days perioperatively (preoperatively, POD 1, POD 3, and POD 6). They found there was no significant change of B cell levels in 46 CRC patients after resection [19]. Additionally, Leung et al. measured B cells at four time-points (preoperatively, POD 1, POD 3, and POD 8). These results, which were similar to those mentioned above, showed no significant difference in 40 rectosigmoid carcinoma patients after resection [26].

Ling et al. reported that in 31 CRC patients the levels of circulating Th17 (defined as IL-17^+^IL-22^−^IFN-γ^−^CD4^+^), Th22 (defined as IL-17^−^IL-22^+^IFN-γ^−^CD4^+^), and IL-17^+^IL-22^+^IFN-γ^−^CD4^+^ T cells were higher on POD 14 than before the operation. However, the percentage of Th1 cells (which not only can produce tumor necrosis factor-beta, interleukin-2, and interferon-gamma, but also activate macrophages) remained unchanged in CRC patients [20].

Colorectal carcinomas are often resected using laparoscopic (LS) or even recently robotic-assisted (RAS) approaches. In this frame, Helvind and colleagues could show that there were no differences between RAS and LS results when regarding the longitudinal changes of WBC. Three studies investigated subsets of lymphocytes in the peripheral blood using flow-cytometry and compared results between RAS, LS, and CS. All three studies reported no significant perioperative changes in the lymphocyte subset cell count between LS, RAS, and CS [19,26,31]. However, T cell activation and cytokine release were different between the groups. These results suggest a less pronounced immunosuppression after minimally invasive surgery [19,26,31].

None of the studies reported on changes between different segments of colonic resection. Most studies did not specify the neoadjuvant or adjuvant treatment. Regarding adjuvant treatment, however, all studies performed cell subset measurements within a timeframe in which no adjuvant chemotherapy is performed. Two studies specified that chemotherapy constituted an exclusion criterion [19,20].

### 3.5. Esophageal Cancer

Changes in immune cells were investigated in 315 patients that received an esophagectomy across three studies (Table 1). Shi et al. reported on regulatory B cells perioperatively, where until POD 7 Bregs remained decreased [22].

The two remaining studies described the differences between minimally invasive and open esophagectomies. Maas et al. reported that WBC could still be elevated in the conventional open surgery group compared to patients who underwent minimally invasive surgery in esophageal cancer patients at POD 7 (Table 1) [32]. This may be related to fewer respiratory infections found after minimally invasive surgery. Tan et al. investigated CD3^+^ cells, CD4^+^ cells, NK cells, and the CD4^+^/CD8^+^ ratio. After minimally invasive esophagectomy these subgroups decreased, then return to preoperative levels on POD 7. After conventional resection, a decrease on POD 1 was noted but it increased more slowly at POD 7 compared to minimally invasive surgery.

### 3.6. Pancreatic Cancer

In pancreatic cancer patients, perioperative immune cell changes were investigated in 73 patients. All patients were operated on with an open conventional technique. As shown in Table 1, the Cdc1 (circulating myeloid dendritic cells 1) count and Cdc1/Cdc2 (circulating myeloid dendritic cells 2) ratio of 20 resected pancreatic cancer patients were increased 12 months postoperatively. There was no significant change in Cdc2 cell counts compared to before surgery. This article concluded that when pancreatic cancer patients did not develop any local recurrence or distant metastasis, Cdc1 count and Cdc1/Cdc2 ratio normalized approximately 12 months after surgery [14].

Takahashi et al. demonstrated in 20 pancreatic cancer patients that no significant differences in NK cell (defined as CD14^−^/CD56^+^) counts were found at POM 12 compared to before surgery [14].

Tezuka et al. reported that the preservation of the spleen resulted in lower WBC level postoperatively. Only 2 out of 53 patients received adjuvant chemotherapy. Therefore, no conclusion about confounding chemotherapy could be drawn [24].

**Table 1:** Summary of included studies. The studies are grouped by following hierarchy: cancer type, follow-up measurement time-points, studied cell types (WBC, NLR, T cells, etc.), and finally, year of publication.

Reference		Study Population				Study Cell Type				Follow-Up Measurement Time-Points	Changing Tendency	Survivals
	Patients	Amount	Study Type	Region	Treatment	Cell Marker	Cell Type	Cell Source	Method			
**Gastric Cancer**												
Takaya, S. et al., 2015 [27]	Gastric Cancer	33	PS	Japan	CS	N/A	Lymphocyte WBC	PB	FAC	POD 1, 3, 7, and POD 30	Lymphocytes: Decreased on POD 1 and then increased, returned to preoperative level on POD 30. Increased on POD 1, then returned to preoperative level on POD 30	N/A
Fujii, K. et al., 2003 [25]	Gastric cancer	20 Including: LS: 10 and CS: 10	PCS	Japan	CS MIS (LS)	Activated NK cell: CD57^+^. Activated lymphocyte: HLA-DR^+^.	WBC Lymphocyte CD3^+^ CD4^+^ CD8^+^ CD57^+^ HLA-DR^+^	PBMC	FAC	POD 1, 3, and POD 7	WBC: increased on POD 1, then returned to preoperative level on POD 7 Lymphocyte: decreased on POD 1, then maintained a low level CD3^+^, CD4^+^, CD8^+^, CD57^+^, and HLA-DR^+^: decreased on POD 1, then returned to preoperative level on POD 7	N/A
Miyatani, K. et al., 2018 [16]	Gastric cancer	280	RS	Japan	CS	N/A	NLR	PB	Cell count	POM 1	POM 1: Both Pre NLR high and POM 1 NLR high Either Pre NLR high or POM 1 NLR high Both Pre NLR low and POM 1 NLR low	5 years survival: 58.1% 75.1% 92.8%
**Hepatocellular Carcinoma**												
Lee W-Ch. et al. 2019, [29]	HCC	19	PS	Taiwan	CS	CD8^+^ CD8^+^ /CD4^+^ CD4^+^/Foxp3^+^ CD33^+^/HLA-DR^-^	T cells T helper Tregs MDSC	PBMC	FAC	PreOP, POD 7 and POM 1	CD4^+^ not altered by resection but CD8^+^ decreased. Tregs were not altered but MDSC were decreased at POM1	OS: 1 y 79.8% 3 y 68.4%
Chen, T. et al., 2012 [28]	HCC	36	PCS	China	CS	CD4^+^CD25^+^CD127^–^ CD19^+^IL-10^+^	Tregs Bregs Lymphocytes	PBMC	FAC	POD 1 and POD 7	Tregs and Bregs: increased on POD 1, especially on POD 7 Lymphocytes: decreased on POD 1 and returned to preoperative level on POD 7	N/A
Peng, W. et al., 2014 [21]	HCC	189	RS	China	CS	N/A	NLR	N/A	N/M	POM 1	POM 1: Increased group: 80 patients Decreased group: 109 patients	Increased group: Poor OS and RFS than NLR decreased group
**Colorectal Carcinoma**												
Helvind, N. M. et al., 2013 [30]	CRC	263 Including LS: 162 and RAS: 101	RS	The Netherlands	MIS (LS and RAS)	N/A	WBC	N/A	N/M	POD 1, POD 2 and POD 3	LS: Pre to POD 1: increased POD 1 to POD 3: decreased RAS: Pre to POD 2: increased POD 2 to POD 3: decreased	N/A
Kubo, T. et al., 2014 [23]	CRC	524	RS	Japan	CS	N/A	NLR	PB	Cell count	POD 1 and POD 3	Divided patients (include Pre, POD 1, and POD 3) into high NLR group and low NLR group	High perioperative NLR score: worse CSS and DFS
Shibata, J. et al., 2015 [19]	CRC	46 Including: RAS: 15; LS: 23; CS: 8	PS	Japan	CS MIS (RAS)	CD3^−^/CD56^+^ CD3^+^/CD8^+^ CD3^+^/CD4^+^ CD3^-^/CD19^+^	NK cells CTL Th B lymphocytes	PB	FAC	POD 1, POD 3, and POD 6	NK cells, CTL, and Th: from Pre to POD 1: decreased, POD 3 and POD 6: increased. B lymphocytes: no significant change	N/A
Ordemann, J. et al., 2001 [31]	CRC	40 Include LS: 20 and CS: 20	RCT	Germany	CS MIS (LS)	N/A	WBC CD4^+^ lymphocytes CD8^+^ lymphocytes CD4^+^/CD8^+^ ratio	PB	FACS	POD 1, 2, 4, and POD 7	WBC: increased on POD 1, then returned to preoperative level on POD 7. CD4^+^ lymphocytes, CD8^+^ lymphocytes and CD4^+^/CD8^+^ ratio: no significant change after surgery	N/A
Leung, K.L. et al., 2003 [26]	CRC	40, Including LS: 20 and CS 20	RCT	Hong Kong	CS MIS (LS)	T cell: CD3^+^ T cell activation: CD3^+^ HLA-Dr^+^ Non-MHC restricted NK cell: CD3^−^CD16^+^CD56^+^ MHC-restricted NK-like cell: CD3^+^CD16^+^CD56^+^. Helper T cell: CD3^+^CD4^+^. Cytotoxic T cell: CD3^+^CD8^+^. NK cell: CD3^−^CD16^+^CD56^+^	T cells T cell activation Non-MHC restricted NK cells MHC-restricted NK-like cells Helper T cells Cytotoxic T cells NK cells WBC Lymphocytes B cells	PB	Cell count	POD 1, POD 3, and POD 8	WBC: increased on POD 1, then returned to preoperative level on POD 8. Lymphocytes, T cells, B cells, Non-MHC restricted NK cells, NK cells, Natural Killer-like T cells, Cytotoxic T cell, Helper T cells, T cell activation: decreased on POD 1, then returned to preoperative level on POD 8	N/A
Wang, Y. et al., 2017 [17]	CRC	7	PCS	China	CS	N/A	T lymphocyte % NK lymphocyte % NKT lymphocyte %	PBMC	FAC	POW 1	POW 1: N.S.	N/A
Ling, L. et al., 2015 [20]	CRC	31	PCS	China	CS	Th1: IL-17-IL-22-IFN-γ^+^CD4^+^. Th17: IL-17^+^IL-22-IFN-γ-CD4^+^. Th22: IL-17-IL-22^+^IFN-γ-CD4^+^	Th1 Th17 Th22 IL-17^+^IL-22^+^IFN-γ-CD4^+^ T cells	PB	FAC	POD 14	Th1%: POD 14: N.S. Th17%, Th22%, and IL-17^+^IL-22^+^IFN-γ-CD4^+^ T cells%: POD 14 were significantly higher than Pre	N/A
**Esophageal Cancer**												
Maas, K.W. et al., 2014 [32]	Esophageal cancer	27 Including CS: 13 and LS: 14	RCT	The Netherlands	CS MIS (LS)	N/A	WBC	PB	N/M	POD 1, POD 3, POD 4, and POD 7	Increased on POD 1, then decreased until to POD 4. But CS group increased on POD 7	N/A
Tan, J.H. et al., 2016 [18]	Esophageal cancer	228 Including: VATS: 52; CS: 176	RS	China	CS MIS (VATS)	N/A	CD3^+^ cells CD4^+^ cells CD8^+^ cells CD4^+^/CD8^+^ ratio NK cells	PB	FAC	POD 1 and POD 7	CD3^+^ cells, CD4^+^ cells, NK cells and CD4^+^/CD8^+^ ratio: VATS: POD 1: decreased, then returned to preoperative level on POD 7 CS: POD 1: decreased, then increased, but POD 7 still lower than Pre CD8^+^ T cells: N.S.	N/A
Shi, J. et al., 2014 [22]	Esophageal cancer	60	PCS	China	CS	CD5^+^CD19^+^	Bregs	PBMC	FAC	POD 1 and POD 7	From POD 1 to POD 7: decreased.	N/A
**Pancreatic Cancer**												
Tezuka, k. et al., 2012 [24]	Pancreatic cancer	53	RS	Japan	CS	N/A	WBC	N/A	N/M	POD 1, 2, 3, 5, 7, POW 2, POM 1, and POM 3	Pre to POD 2: increased, then decreased until POM 3	N/A
Takahashi, K. et al., 2006 [14]	Pancreatic cancer	20	PCS	Japan	CS	CD11c^+^ DCs CD11c^−^ DCs CD14^−^/CD56^+^ CD3^+^/CD4^+^ CD3^+^/CD8^+^	Cdc1 Cdc2 NK cells CD4^+^ T lymphocytes CD8^+^ T lymphocytes Cdc1/cDC2 ratio	PB	FAC	POM 12	cDC1 and cDC1/cDC2 ratio increased in POM 12. cDC2: N.S. CD4^+^ T lymphocytes, CD8^+^ T lymphocytes, and NK cells: no significant change in POM 12	cDC1 count and cDC1/cDC2 ratio normalized in POM 12: no obvious local recurrence or distant metastasis

**Abbreviations:** HCC: Hepatocellular carcinoma; CRC: Colorectal cancer; Bregs: Regulatory B cells; Tregs: Regulatory T cells; Th: Helper T cells; WBC: White blood cell; NK: Natural killer; NKT: Natural Killer T; NLR: Neutrophil to lymphocyte ratio; CTL: Cytotoxic T lymphocytes; Cdc1: Circulating myeloid dendritic cells 1; Cdc2: Circulating lymphoid dendritic cells 2; N/A: Data not found; N/M: No experimental methods; N.S.: Data found but have no significance; OS: Overall survival; RFS: Recurrence-free survival; CSS: Cancer-specific survival; DFS: Disease-free survival; PB: Peripheral blood; PBMC: Peripheral blood mononuclear cells; FAC: Flow cytometry analysis; FACS: Fluorescence-activated cell sorting; Pre: Preoperation; POD: Postoperation day; POW: Postoperation week; POM: Postoperation month; RAS: Robot-assisted surgery; RCT: randomized controlled trial; RS: retrospective study; PCS: Prospective case control study; PS: prospective study; LS: Laparoscopic surgery; CS: Conventional surgery; VATS: Video-assisted thoracoscopic surgery; MIS: Minimally invasive surgery.

### 3.7. Cell Subsets across Entities

As seen in Table 1, a total of 10 (55.56%) studies investigated T cells. Three of them observed a significant reduction in T cell (defined as CD3^+^) [18,25,26,29] counts and activated T cell (defined in this publication as CD3^+^HLA-DR^+^) [26] counts after surgery, compared with before surgery. In all of the above-mentioned reports T cell levels recovered to the preoperative level about one week after surgery (POD 7).

Seven (38.89%) reports described CD4^+^ cells and CD8^+^ cells. Most reports (57.14%) showed that these two kinds of cells decreased on the first postoperative day compared with preoperative levels, and gradually recovered to preoperative level by postoperative week one [18,19,25,26,31] or month 12 [14].

Four (22.22%) studies provided information about B cells. Two studies reporting on CRC showed no differences regarding Bcells levels perioperatively [19,26]. Two studies reported results regarding regulatory B cells (Bregs). One study by Shi et al. demonstrated that there was no significant difference in the percentage of Bregs (defined as CD5^+^CD19^+^ cells) between 60 patients with esophageal cancer before CS and POD 1. However, a significant reduction of Bregs was observed in esophageal cancer patients seven days after tumor resection compared with the counts before the surgery and POD 1 [22]. Conversely, Chen et al. also examined Bregs (defined as CD19^+^IL-10^+^ cells) pre- and postoperatively in HCC patients. The frequency of peripheral Bregs significantly increased after tumor surgery at POD 7 [28].

Three (16.66%) studies showed that the postoperative day 1 level of NK cells [18,19] and their subsets (Non-MHC restricted NK cells (defined as CD3^−^CD16^+^CD56^+^) and MHC-restricted NK-like cells (defined as CD3^+^CD16^+^CD56^+^)) [26] were significantly lower in PB measurements compared to before the operation. However, these cell counts almost returned to the preoperative level about one week after surgery (POD 7).

## 4. Discussion

Surgery represents the mainstay of curative treatments of gastrointestinal cancer [33,34]. However, for the body, surgery itself and its trauma may cause changes in the immune system, which can affect the immediate and long-term prognosis of patients [6,8]. This systematic analysis describes the relationship between the distribution of PB immune cells in patients with digestive system resections during the perioperative period and their prognosis. This overview includes WBC count, lymphocytes count, NLR, CD4^+^ T cells, CD8^+^ T cells, Th, B cells, NK cells, DCs, and immunosuppressive cells, for instance, Tregs, Bregs, and MDSC. Therefore, this work represents the most comprehensive systematic analysis about this topic to date.

WBC count has commonly been used as an indicator for detection of inflammation in clinical practice [35]. The common causes of WBC elevation after surgery are stress response caused by surgical trauma, and of course, postoperative infections [36]. The increase caused by the stress response is transient, and mostly returns to the preoperative level within one week after surgery. Maas et al. pointed out that compared with minimally invasive surgery, traditional surgery has a relative large trauma and slower recovery, which in turn might cause a higher increase of WBC [32]. WBC, however, include all Leukocytes which might have immune activating and immune suppressing functions. Therefore, it is not clear what effect a rise in WBC might have. A more detailed look can be obtained with the differential blood cell count [37]. The overall trend is similar to that of WBC, in which the lymphocyte counts decreased significantly after surgery, and then gradually returned to normal levels. Within the differential blood cell count also neutrophil leukocytes are counted, which opens the possibility for obtaining an impression of the balance of systemic inflammation and immune response after surgery [21]. The results indicate that high NLR before and/or after surgery are associated with a poor prognosis. Postoperative systemic inflammatory response leading to increased neutrophils shifts the balance against lymphocytes. Neutrophils are suspected to have an immunosuppressive function in the presence of the tumor [38]. Since most lymphocytes consist of T cells, a decrease in their amount after resection should be found accordingly. In this analysis lymphocytes showed a downward trend after surgery. Over time, the body’s immune response is restored, and neutrophil levels decrease, leading to a gradual return of T cell levels to the preoperative level. NK cells develop on a similar direction as T cells. They also gradually return after approximately 7 days. There is mounting evidence that this temporary decrease is less after minimally invasive surgery [18,39]. Conversely, T cells with immunosuppressive effects increase after surgery [20]. Combining the immunological effects of the above-mentioned cells, the possible explanation is that the immunosuppressive state caused by surgery is more conducive to cells with immunosuppressive function, causing the levels of the above-mentioned immune cells to increase after surgery.

The picture is less clear when looking at the humoral immunity. In general, the humoral immunity is less dynamic and less susceptible to immune modulatory agents such as prostaglandins and proinflammatory cytokines [40]. This might be a reason why no changes in B cell distribution could be detected in the literature [19,26]. When looking at immunosuppressive Bregs, Chen et al. reported that the levels of cells increased postoperatively, especially peaking at 7 days postoperatively [28]. Conversely, the results of Shi et al. showed that Bregs showed a downward trend at one week after surgery [22]. One possible explanation for the results of Chen et al. is that the increase of Tregs and Bregs after the operation is caused by immunosuppression after resection. The opposite results obtained by Shi et al. were interpreted by the authors as an effect of removing the tumor lesion, which might have had an immunosuppressive effect. Both effects might be present and differently pronounced dependent on tumor type, size, and differentiation, or for example, liver cirrhosis, which also can lead to immunosuppression [41,42].

DCs are the most important antigen-presenting cells and play an important role in the initiation and regulation of the immune response [14]. As reported in one publication, peripheral blood Cdc1 levels, and Cdc1/Cdc2 ratios increased in patients with pancreatic cancer one year after surgery, but Cdc2 levels did not change significantly. Additionally, compared with patients with local recurrence and metastasis after surgery, the level of Cdc1 and the ratio of Cdc1/Cdc2 in patients without recurrence and metastasis returned to normal one year after surgery. A possible explanation might be that although surgery itself is traumatic and can cause immunosuppression, surgery can remove the tumor lesion and greatly alleviate the burden of anti-tumor immunity.

This systematic analysis has limitations that are inherent to the complexity and longitudinal experimental setting that is required for such investigations. Although the amount of literature was limited and the follow-up time was inconsistent, it contains the most common tumor diseases of the digestive system and a relatively comprehensive amount of immune cell types. Some of the results opposed each other, which reflects a heterogeneity within the large group of gastrointestinal system tumors. Furthermore, besides the entity, many other factors may influence the distribution of immune cells. Miyatani et al. suggested that the adjuvant chemotherapy might influence NLR. However, they further report that most gastric cancer patients that received adjuvant chemotherapy received it 4–6 weeks after the operation. This timepoint is after the recovery of the surgical trauma [16]. Another confounding factor could be chronic inflammation, which was investigated by Chen et al. in HCC patients. In their work, they report that chronic hepatitis B patients generally had lower levels of Tregs but the longitudinal trend of the surgical resection was the same between hepatitis and non-hepatitis patients [28]. The effect of hepatitis or general chronic inflammation cannot be underestimated. Therefore, in our experimental work we have opted to investigate non-HBV/non-HCV patients to mitigate these biases [42]. Lastly, the invasiveness of the operation seems to be influencing the amount of immunosuppression deriving from the trauma. In colorectal carcinoma, laparoscopically operated patients showed a similar trend but it was less pronounced as compared to conventional surgery [26]. These differences underline the many confounding factors when investigating the longitudinal changes of immune cells perioperatively. Besides the differences, many similarities could also be detected. This strengthens the assumption of a general immune modulation because of the trauma of the operation.

In light of the dawn of novel immunotherapeutic agents, perioperative immune cell changes are an emerging field. Since the fundamental mechanisms of anti-tumor effects are fundamentally different to traditional chemotherapy, old paradigms such as the need for a neoadjuvant and adjuvant treatment might not be necessary anymore. However, immunotherapy can cause adverse events such as pneumonitis, hepatic failure, or even anaphylactic reactions. At the moment there are no results on the safety of the use of, e.g., immune checkpoint inhibitors during the immediate perioperative phase.

In summary, the trauma caused by the operation might lead to a decrease in the level of immune cells in the body. Compared with open surgery, minimally invasive surgery probably has less transient immunosuppression. The aggregation of these, in part, contradictory results show that comprehensive understanding of the distribution of immune cells in the PB during the perioperative period and the relationship with the short and long-term prognosis of patients should be further explored in translational studies.

## Data Availability

Original data will be made available upon reasonable request.

## Abbreviations

Bregs	Regulatory B cells
CD	Cluster of differentiation
CRC	Colorectal cancer
DCs	Dendritic cells
HCC	Hepatocellular carcinoma
MDSC	Myeloid derived suppressor cells
NK	Natural killer cells
NLR	Neutrophil-lymphocyte ratio
PB	Peripheral blood
POD	Postoperation day
POM	Postoperation month
Th	Helper T cells
Tregs	Regulatory T cells
WBC	White blood cells
